# A Novel Case of Cerebral Diplopia Secondary to a Posterior Parietal Cortex Ischemic Infarct: Proposal of a Mechanism of Generation of Polyopia Due to Convergence Insufficiency

**DOI:** 10.7759/cureus.12962

**Published:** 2021-01-28

**Authors:** Hassan Kesserwani

**Affiliations:** 1 Neurology, Flowers Medical Group, Dothan, USA

**Keywords:** transient visual loss, stroke

## Abstract

All reported cases of cerebral polyopia in the literature are associated solely with occipital lobe disease, as seen with ischemic infarcts, migraine, trauma, and epilepsy. To our knowledge, this is the first case of polyopia associated with posterior parietal cortex (PPC) ischemic infarct to be reported in the literature. Previous hypotheses about mechanisms of polyopia have included cortical spreading depression, the vague idea of abnormal visual synthesis, and the holographic or holonomic brain theory. We propose a new mechanism due to dysfunction of the network from the PPC $\rightarrow$ frontal eye field (FEF) $\rightarrow$ paramedian midbrain and pontine reticular formation leading to convergence insufficiency, which leads to horizontal diplopia. The evidence ranging from tracer studies in macaque monkeys to functional MRI (fMRI) studies in patients with convergence insufficiency is presented to bolster our hypothesis. In the process, we also briefly review the neural pathways of convergence.

## Introduction

We have previously described in Cureus a case of cerebral diplopia (polyopia) due to an ischemic infarct of the striate cortex, visual (V1), and adjacent visual association cortex (V2) [1]. Polyopia is defined as the duplication of images (doublets, triplets, and more) in the absence of a refractive error, fascicular cranial nerve disease, orbital pathology, or brainstem disease.

Polyopia has been described before with occipital lobe disease due to trauma, ischemia, epilepsy, and migraine [2-5]. We also reviewed potential mechanisms including cortical spreading depression and abnormal visual synthesis, and we posited a new hypothesis: the holonomic brain theory [1].

For the first time, we report a case of cerebral diplopia arising from an ischemic infarct of the posterior parietal cortex (PPC). In the Discussion section, we propose a mechanism for the generation of cerebral diplopia due to a PPC ischemic infarct based on the connection of the PPC with the frontal eye field (FEF) and the latter's projection to the midbrain reticular formation and paramedian pontine reticular formation (PPRF). We propose that the diplopia arises from convergence insufficiency. We have previously outlined visual object recognition and visual-spatial motion and the role of the dual ventral and dorsal visual pathways, which we briefly outline in the Discussion section [6].

## Case presentation

We present the case of a right-handed 90-year-old healthy and independent man who developed the sudden onset of isolated, painless, and intermittent horizontal diplopia. The separation of images had no gaze preference and was triggered by staring, such as watching television or looking at a close object. He denied the presence of any other brainstem symptoms such as vertigo, dysarthria, dysphagia, symmetric or asymmetric sensory symptoms, or ataxia. There was an absence of fatiguability in the afternoon.

His past medical history was unremarkable and he took no prescription medications. On examination, his blood pressure was 121/78 mmHg, and he had a heart rate of 82 beats per minute. He was 6 feet tall and weighed 140 pounds. His gait was wide-based and he used a cane. He was able to tandem-walk with assistance. Romberg sign was absent. Cognitively, he was sharp with focused and non-hesitant rapid response to questions. His speech was fluent without dysarthria, but with mild hypophonia.

Cranial nerve examination revealed full ocular motility in all directions without ptosis or pupillary abnormalities. Visual fields were full to confrontation without visual neglect by sequential visual field testing. The rest of the cranial nerve function was normal. Power was normal and deep tendon reflexes were surprisingly lively but without spasticity. Coordination testing revealed a mild intention tremor in both upper extremities. Sensory examination revealed a mild loss of vibratory sense in his big toes.

**Figure 1:**
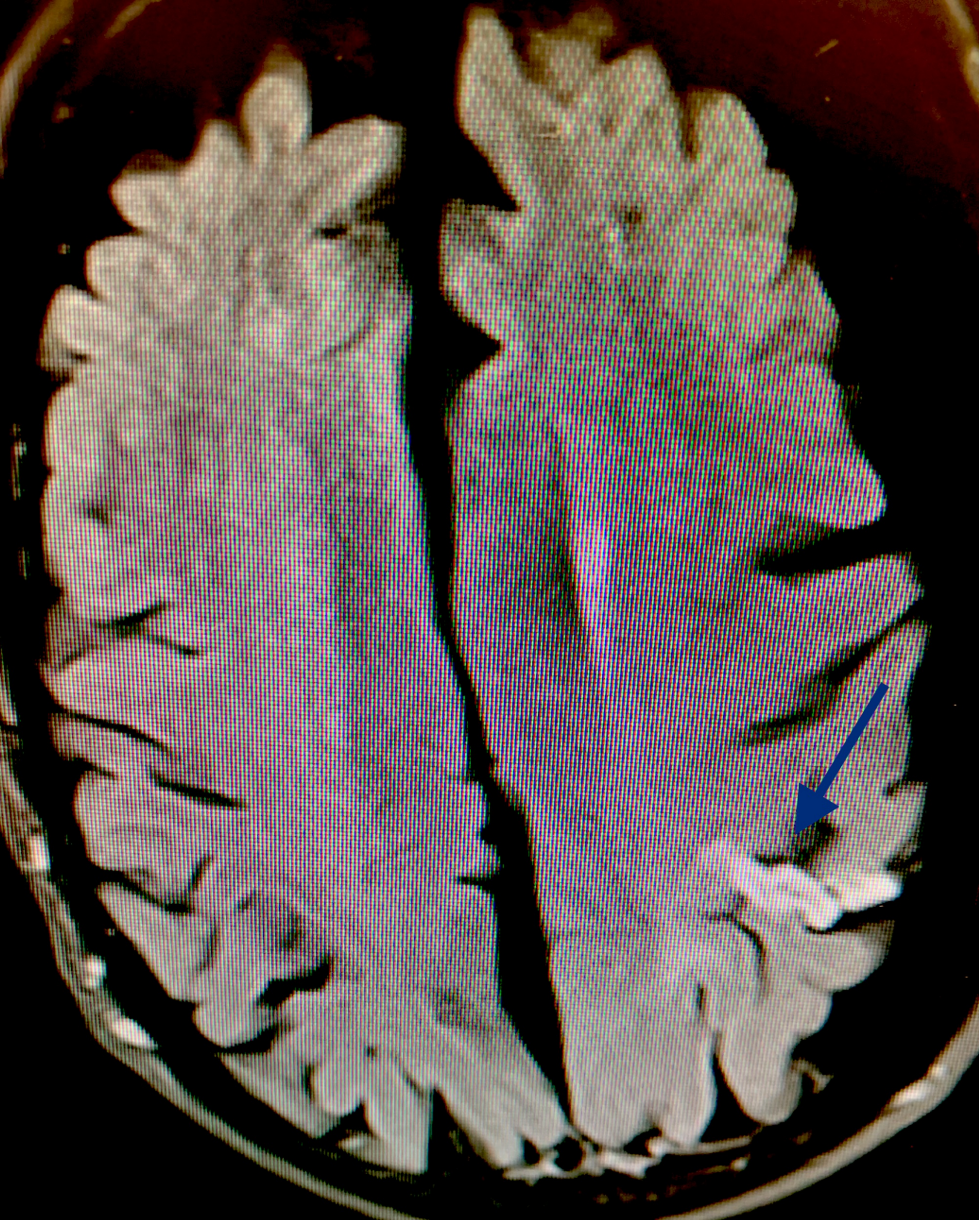
MRI-DWI showing left posterior parietal lobe cortical ischemic infarct (blue arrow) MRI: magnetic resonance imaging; DWI: diffusion-weighted imaging

An MRI study with diffusion-weighted imaging (DWI) sequence revealed a left posterior parietal cortical ischemic infarct (Figure 1).

**Figure 2:**
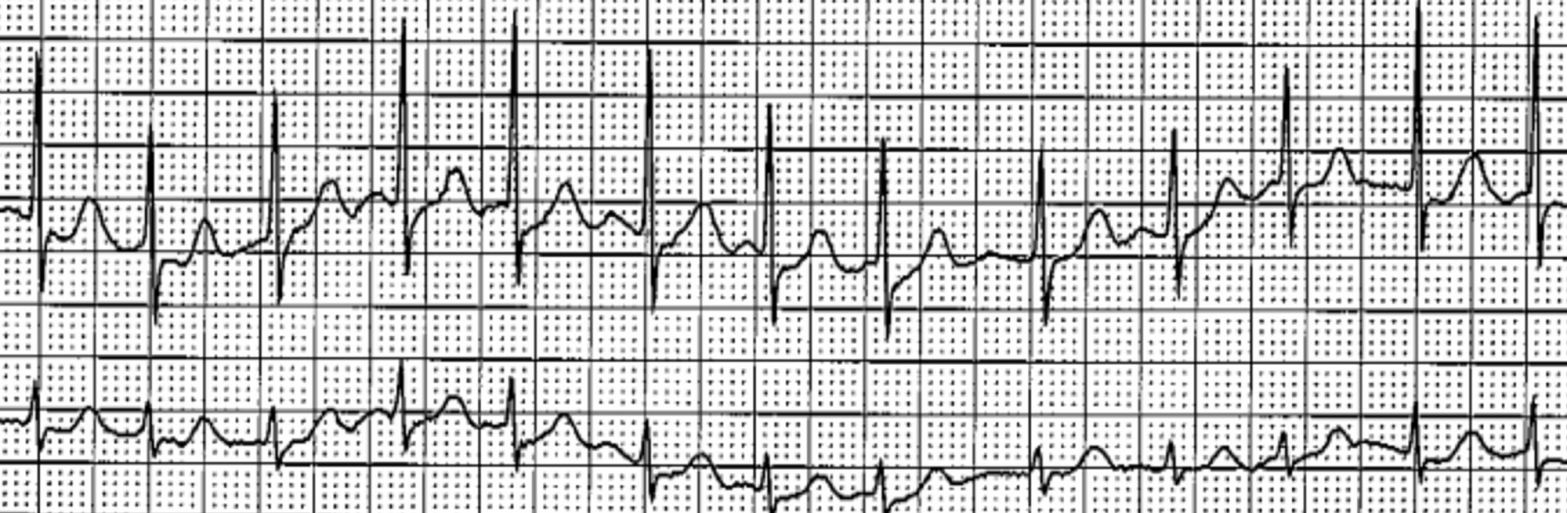
Cardiac rhythm strip demonstrating atrial fibrillation with a heart rate of 132

A cardiac event monitor revealed paroxysmal atrial fibrillation one week after the onset of the cerebral ischemic infarct (Figure 2).

A carotid duplex scan and a transcranial Doppler study of the cerebral circulation revealed no flow-limiting vascular lesions. Due to the embolic nature of the cortical ischemic infarct, demonstration of atrial fibrillation, and his advanced age of 90 years, the patient was started on low-dose apixaban 2.5 mg twice daily. He refused a referral to a cardiologist or an optometrist/ophthalmologist despite encouragement to do so.

## Discussion

The dual visual pathway begins in the striate cortex and subdivides into the "what" pathway, a ventral stream, running along the occipitotemporal lobes and is involved in visual perception by identifying objects, and the "where" pathway, which ends at the PPC allowing spatial localization of objects [7].

Dysfunction of the ventral stream can lead to prosopagnosia (inability to recognize and identify faces), impaired object recognition and identification, and semantic dementia. Dysfunction of the dorsal stream has been associated with the Zeitraffer phenomenon (perception of objects moving slower), optic ataxia (visually directed dysmetria), asimultagnosia (inability to synthesize a visual scene as in fragmented visual processing of a scene), impaired subitization (difficulty visually estimating the number of objects in a small cluster of objects), and akinetopsia (frozen scenes) [8].

How are the dorsal and ventral streams combined for perception and action? Tracer injection studies in macaque monkeys have demonstrated the projection of these pathways into the FEF, where purposeful visually-directed saccades are generated. The visual-orienting responses from the low-visual-acuity peripheral retina are generated by high-amplitude saccades in the medial FEF and direct the eyes to an object in the periphery of vision for spatial vision. Low-amplitude saccades are generated by the lateral FEF and involve high-acuity foveal vision for both spatial and object vision. The FEF selects the appropriate saccadic amplitude, small or large, and is quite unique in receiving projections from the PPC [9].

In humans, the FEF is located at the distal end of the middle frontal gyrus, immediately adjacent to the pre-central sulcus. The FEF is present in all primates and generates the voluntary command for saccades [10]. The FEF controls saccadic eye generation via four pathways [11]: 

$1)$ Projection to the striatum, which controls saccadic latency and speed

$2)$ Projection to the superior colliculus (part of dorsal stream trajectory), which regulates attention to stimuli

$3)$ Projection to pontine nuclei and thence to the cerebellum, which controls saccadic speed and accuracy

$4)$ Projection to PPRF, the brainstem horizontal saccade generator.

**Figure 3:**
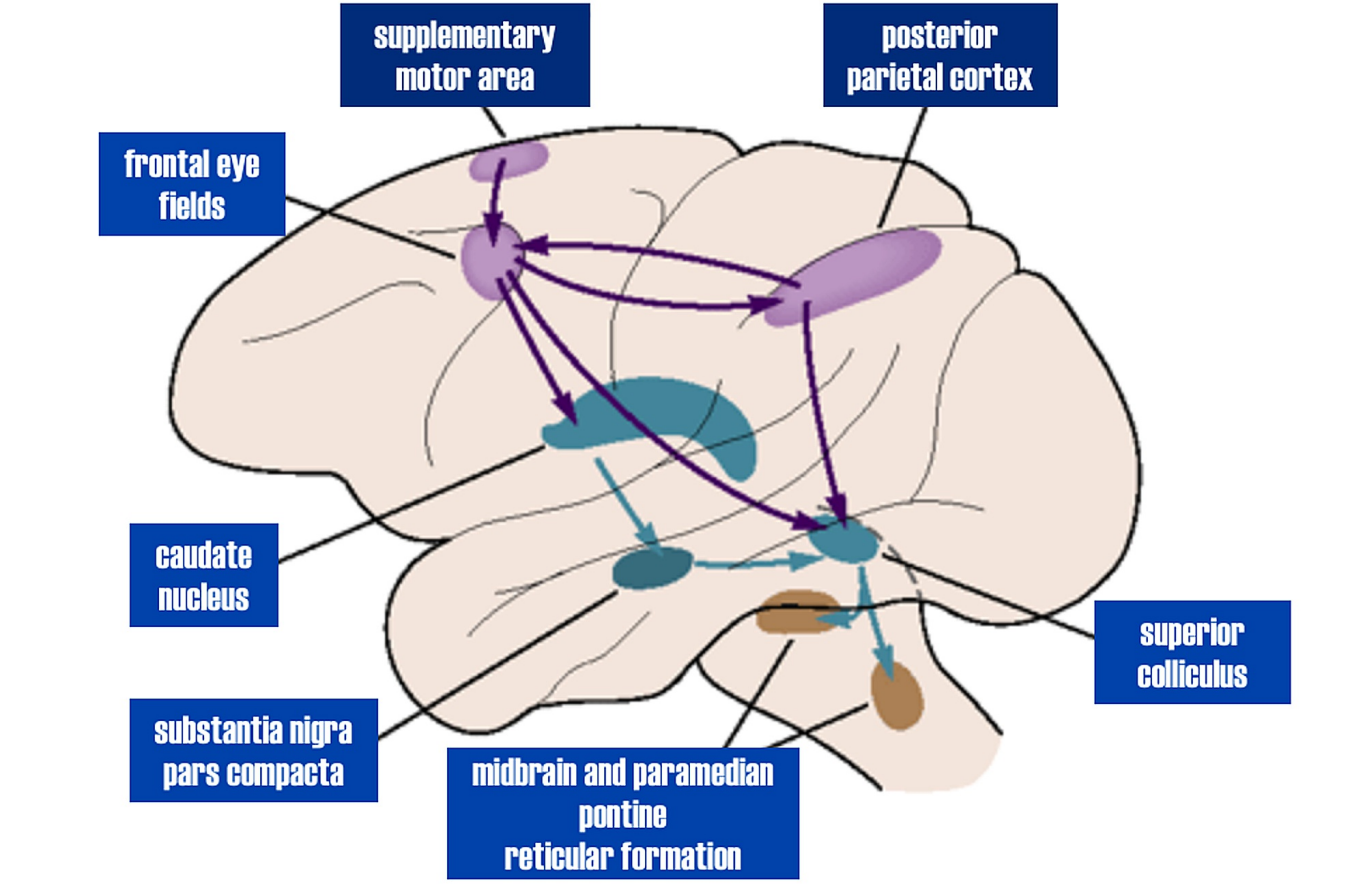
Diagram demonstrating projections of FEF into midbrain reticular formation and PPRF, superior colliculus, and substantia nigra pars compacta FEF: frontal eye field; PPRF: paramedian pontine reticular formation

These pathways are demonstrated below (the projection to the cerebellum is omitted) (Figure 3).

There are also feedback loops with the thalamus. The FEF receives thalamic input from the mediodorsal and ventro-anterior nuclei of the thalamus. These nuclei in turn receive input from the superior colliculus, substantia nigra pars reticulata (SNpr), and the dentate nucleus of the cerebellum. The interesting point to note is that the SNpr tonically inhibits the superior colliculus. Prior to the initiation of a voluntary saccade, this tonic inhibition is halted briefly and a motor command from the FEF goes to the PPRF, thereby generating a saccade [12].

**Figure 4:**
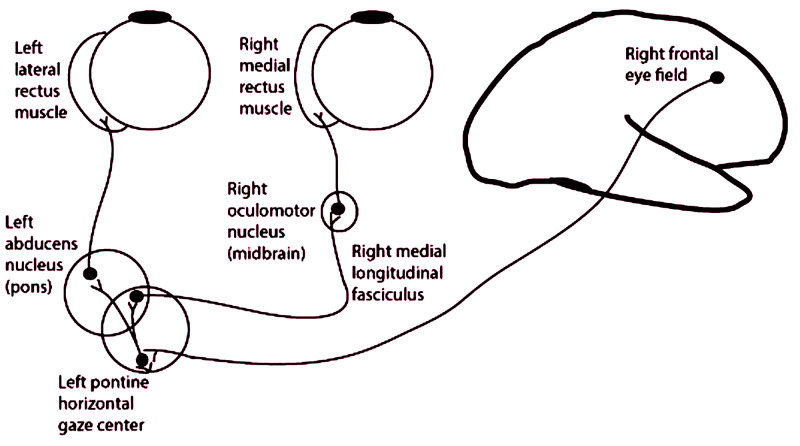
Generation of horizontal gaze – projection from right FEF to left PPRF and thence to ipsilateral abducens nucleus and contralateral oculomotor complex FEF: frontal eye field; PPRF: paramedian pontine reticular formation

The PPRF generates horizontal saccades by projecting to the ipsilateral abducens motor nucleus (ipsilateral abduction) and contralateral oculomotor complex (contralateral adduction) (Figure 4) [13].

However, the medial longitudinal fasciculus (MLF) is not involved in convergence eye movement. Lesions of the MLF do not lead to convergence insufficiency.

The cortical signal for convergence eye motion begins in the occipital (striate and para-striate cortex) and posterior parietal lobes and projects to the FEF. From here, the signal travels to the thalamus (pulvinar) and basal ganglia. The convergence eye command then reaches the midbrain reticular formation and the vicinity of the medial rectal nuclei adjacent to the oculomotor nucleus. Here we find tonic cells (vergence angle), burst cells (vergence velocity), and tonic-burst cells (vergence angle and velocity). This matrix of cells is pre-motor neurons that are known as the "near-response cells". The firing rates of these cells is proportional to the vergence angle and velocity [14,15]. Other areas involved in the generation of convergence eye movements include the PPRF, the nucleus reticularis tegmenti pontis (NRTP), and the interposed and fastigial nuclei of the cerebellum [16].

**Figure 5:**
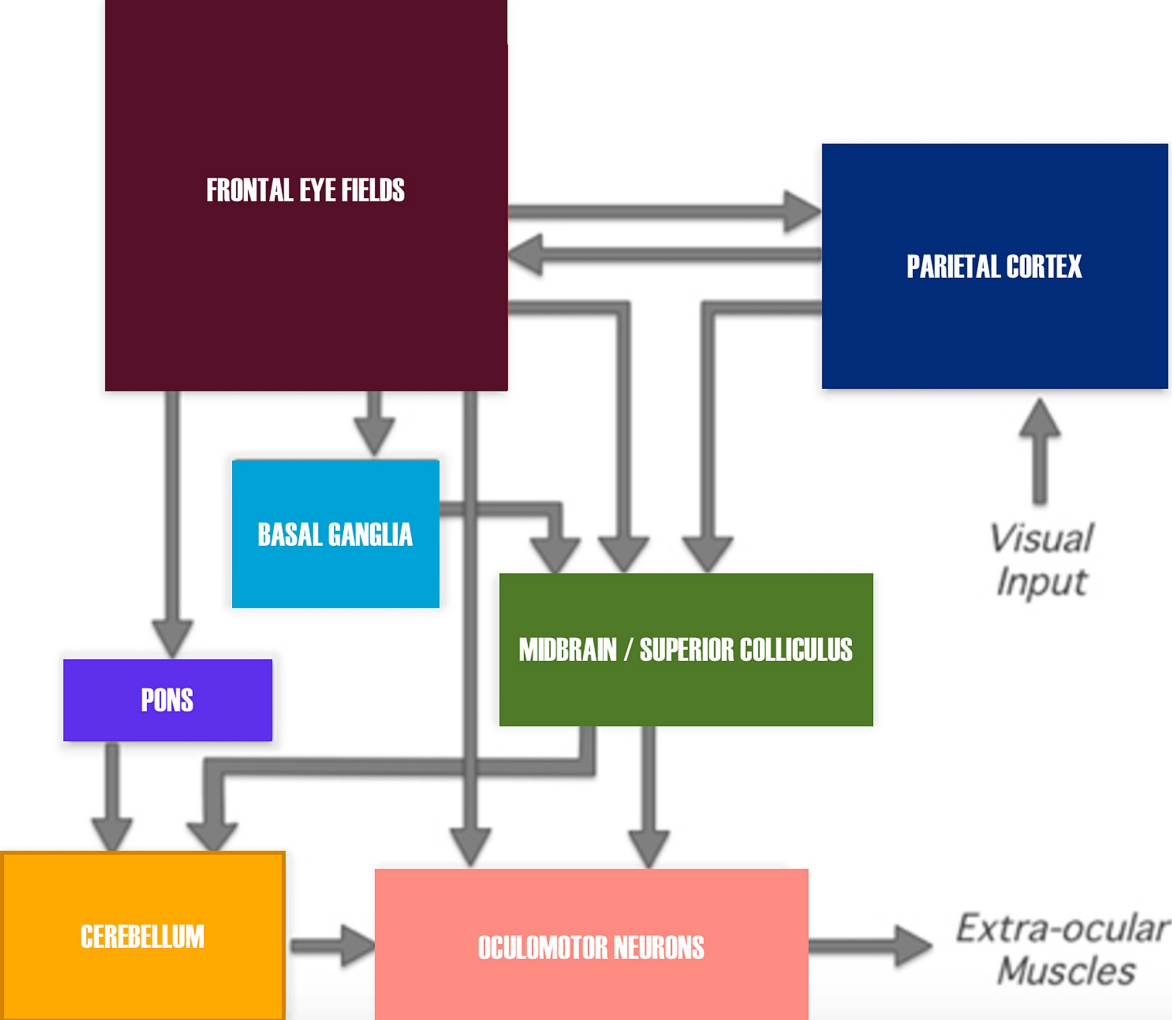
Convergence pathway from the parietal lobes to frontal eye fields to brainstem reticular formation The convergence pathway bypasses the medial longitudinal fasciculus

The final common pathway for convergence eye motion is the Edinger-Westphal nucleus (EWN), a parasympathetic ganglion, which is involved in pupillary constriction and convergence. The EWN receives synapses from the fastigial nucleus of the cerebellum, superior colliculi, and the pre-tectal midbrain [17]. These pathways are illustrated below (Figure 5).

Convergence insufficiency is a disorder of near-object viewing, leading to a recession of near-point convergence. It can lead to diplopia, eye-strain, and blurring of vision. Convergence insufficiency can be seen with aging, traumatic brain injury, and neurodegenerative disorders such as Parkinson's disease and progressive supranuclear palsy (PSP) [18].

A remarkable functional MRI (fMRI) study measured cerebral blood flow in convergence insufficiency subjects compared to normal controls. In the convergence insufficiency subjects, there was diminished blood flow in the PPC, FEF, and cerebellar vermis, which improved with vergence training [19].

We will need one more idea, the concept of diaschisis or functional deafferentation. An ischemic region may be rendered electrically silent, and nodes upstream and upstream to the ischemic lesion are also momentarily electrically silenced. In our case, the PPC ischemic infarct, which projects to the FEF, may silence the FEF. This phenomenon cascades down into the midbrain reticular formation and PPRF. The electrical silence of the PPC, FEF, and midbrain reticular formation and PPRF may lead to convergence insufficiency and horizontal diplopia. Given the experimental data and imaging studies, this is the most plausible explanation [20]. Unfortunately, the patient's refusal to visit with an optometrist did not allow us to prove our theory in confirming convergence insufficiency, which requires sophisticated optometric testing. Future research should involve fMRI studies of patients with PPC ischemic infarcts.

## Conclusions

To the best of our knowledge, this is the first report of cerebral diplopia associated with a posterior parietal cortex ischemic infarct. We presented the evidence to support dysfunction of the network from the PPC $\rightarrow$ FEF $\rightarrow$ midbrain and PPRF leading to convergence insufficiency and subsequent horizontal diplopia. We also engaged in a review of the convergence neural pathways.
